# Identification of Important Nodes in Directed Biological Networks: A Network Motif Approach

**DOI:** 10.1371/journal.pone.0106132

**Published:** 2014-08-29

**Authors:** Pei Wang, Jinhu Lü, Xinghuo Yu

**Affiliations:** 1 School of Mathematics and Information Sciences, Henan University, Kaifeng, China; 2 Institute of Systems Science, Academy of Mathematics and Systems Science, Chinese Academy of Sciences, Beijing, China; 3 School of Electrical and Computer Engineering, RMIT University, Melbourne, Victoria, Australia; University of South Australia, Australia

## Abstract

Identification of important nodes in complex networks has attracted an increasing attention over the last decade. Various measures have been proposed to characterize the importance of nodes in complex networks, such as the degree, betweenness and PageRank. Different measures consider different aspects of complex networks. Although there are numerous results reported on undirected complex networks, few results have been reported on directed biological networks. Based on network motifs and principal component analysis (PCA), this paper aims at introducing a new measure to characterize node importance in directed biological networks. Investigations on five real-world biological networks indicate that the proposed method can robustly identify actually important nodes in different networks, such as finding command interneurons, global regulators and non-hub but evolutionary conserved actually important nodes in biological networks. Receiver Operating Characteristic (ROC) curves for the five networks indicate remarkable prediction accuracy of the proposed measure. The proposed index provides an alternative complex network metric. Potential implications of the related investigations include identifying network control and regulation targets, biological networks modeling and analysis, as well as networked medicine.

## Introduction

Complex network theory and its applications have been popular topics in recent years [1]–[8]. Many real-word systems can be described by complex networks and investigated through complex network theory, such as social systems, biological systems. Gene regulatory networks, signal transduction networks, neural networks, protein-protein interaction networks, metabolic networks are typical biological networks, which have been extensively investigated in related areas [9]–[15].

Complex networks consist of nodes and edges. An edge denotes the interaction between two nodes, which can be directed or undirected. Many biological networks are directed ones. For example, in gene regulatory networks, nodes represent genes or transcription factors, edges represent the interactions between transcription factors and the regulated genes, or between transcription factors. Over the last decade, identification of important nodes in complex networks has been an intriguing topic [16]–[32]. For example, in social networks, provided that one knows which nodes are the most important ones, one can control these nodes in priority to prevent the spread of infectious diseases [16]. However, it is still a challenge to determine which nodes are important in a complex network. Traditionally, degree is frequently used to characterize the importance of a node [1]–[3], [6]–[8], [16]. The other indexes include the betweenness [19], closeness [1], k-shell [7], principal component centrality [17] based on adjacency matrix of the network, semi-local centrality [20], motif centrality [25]–[30], PageRank [21] and others therein.

For undirected networks, some researchers believe that the most connected nodes are the most influential [1]–[3]. But recently, Kitsak et al. [31] investigated the spreading dynamics on four real-world complex networks. They found that for networks with a single initial spreader, k-shell can predict the outcome of spreading dynamics more reliably than degree and betweenness. Following, Chen et al. [20] proposed a semi-local centrality, which considers the degrees of both the nearest and next nearest neighbors of a node. The semi-local centrality can more effectively characterize influential spreaders in complex networks than the degree and betweenness. Recently, following the method in [31], we identified influential spreaders in artificial random, small-world and scale-free networks. Some general conclusions have been obtained [32].

However, though there are numerous results reported on undirected networks, few results have been reported on directed biological networks [25]–[30]. In 2004, Sporns et al. [27] proposed a concept of motif fingerprint in brain networks, which counts the appearances of each node in network motifs with a given size as a measure. In 2007, based on the motif fingerprints and some of the other centrality measures, Sporns et al. [28] investigated the identification and classification of hubs in some brain networks. Also in 2007, based on the concept of network motif, Koschützki et al. [25], [26] proposed some new motif-based measures for gene regulatory networks. They took the occurrences of each node in the 3-node feed-forward loop (FFL) as a measure, after further considering the direction of each edge, another two extended measures were proposed. Interesting results on finding the global regulators in the gene regulatory network of E. coli have been reported.

In this paper, based on the occurrences of each node in all 2-node, 3-node and some 4-node network motifs and the PCA, we aim at developing a new method to characterize node importance in directed biological networks. To evaluate the performance of the new index, the in-degree, out-degree, total degree, PageRank, motif centrality and betweenness are considered to compare with the proposed one. Investigations on five real-world biological networks illustrate the performance of the proposed measure.

## Materials and Methods

### Network Motifs and Motif Detection

**Figure 1:**
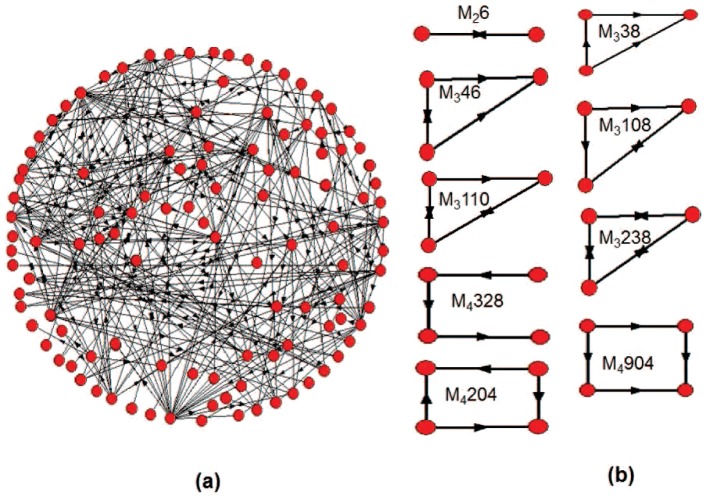
A real-world biological network and some network motifs. (a) A Drosophila developmental transcriptional network with 119 nodes and 306 directed edges. (b) Some representative 2, 3 and 4-node motifs.

In 2002, Alon et al. proposed the concept of network motif, which is defined as subgraph that appears in a network significantly more than in randomized ones [9], [11]–[13]. Network motifs are building blocks of complex biological networks [11]. Functions of some motifs have been extensively investigated. For example, for the FFLs, researchers have theoretically and experimentally found its functional and structural advantages [33]–[37]. Two-node motifs include the double negative feedback loop, double positive feedback loop, and that with auto-activation or repression loops [38], [39]. Three-node motifs include the FFLs, the repressilator and so on [39], [40], with some of them as shown in Fig. 1. Fig. 1 (a) shows the Drosophila developmental transcriptional networks with 119 nodes and 306 directed edges [13]. Fig. 1(b) shows some representative 2, 3 and 4-node motifs. The motifs in Fig. 1(b) is coded as *M_i_j*, where the subscript *i* denotes the size of the motif, *j* is the motif ID number, which is a decimal number that transformed from the adjacency matrix of the motif (For details, one can refer to Mfinder tool guide [41]).

To detect network motifs, Milo et al. [11] scanned all possible *i*-node subgraphs in a network and its randomized counterparts, and defined network motifs as subgraphs for which the probability of occurrences in the real network are greater than that in randomized ones. Since 2002, many motif detection algorithms and softwares [41]–[46] have been developed. For example, gSpan [43], Mfinder [41], FANMOD [45], Mavisto [46] and mDraw (http://www.weizmann.ac.il/mcb/UriAlon). In the following, we use mDraw to detect network motifs. For each network, we generate 100 randomized networks. Number of a subgraph in the real-world network is denoted as *N*_real_. The average number in 100 random networks is denoted as *N*_rand_, with standard deviation denoted by *S*_d_. The *Z*_score_ measures the significance of the subgraph [11], which is defined as *Z*_score_ = (*N*_real_−*N*_rand_)/*S*_d_. Another index *U* is defined as the number of times a subgraph appears in the investigated network with distinct sets of nodes. In this paper, subgraphs with *Z*_score_≥2, *U*≥4 and *N*_real_−*N*_rand_≥0.1*N*_rand_ are identified as motifs.

### A new measure of node importance based on network motifs

Based on network motifs, we develop a new measure to characterize node importance in directed biological networks. Biological networks consist of some motifs, which act as functional units of the complex networks. For example, it has been found that the FFLs play functional roles in gene regulatory networks, such as an incoherent FFL can act as a fold-change detector [9], [35]. Some other 3-node motifs and the 4-node bi-fan motif *M*_4_204 are also found to play functional roles in biological systems [9], [14]. Therefore, nodes that frequently involved in network motifs may be more important. If a node involves in several different types of network motifs, then this node may potentially have multi-functional roles. Keeping the idea in mind, some related measures have been proposed to investigate the biological networks [25]–[30]. We noted that in some works, network motifs are treated as subgraphs, such as the works of Rubinov et al. [30] and Wuchty et al. [42].

Hereinafter, different from the works in [25]–[30], based on all 2, 3 and some 4-node motifs in directed networks, we propose a new integrative measure. Specifically, suppose we have a directed network with *n* nodes, and there are totally *m* types of 2, 3 and 4-node motifs. We denote the occurrences of node *i* in the *j*-th type of motif as *u_ij_*, *i* = 1,…, *n*, *j* = 1,… *m*. Then, we derive a matrix *A* = (*u_ij_*)*_n_*_×*m*_ for the network. In real-world networks, the importance of different types of motifs are varied. Therefore, we endow each motif with a weight *w_j_*, *j* = 1, 2,…,*m*, where 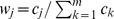, here, *c_k_*(*k* = 1, 2,…,*m*) denotes the number of the *k*-th type of motif. Subsequently, we derive a revised matrix 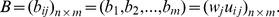


Based on *B* and the idea of the PCA [47]–[49], we construct the following index to obtain node importance score:
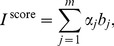
(1)where 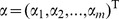 are parameters to be determined. The best index vector *I* ^score^ should have high distinguish ability among different nodes. Therefore, the variance of *I* ^score^ should be as large as possible. Taking 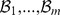 as random variables, which represent the weighted counts of a node in the *m* types of motifs. For a certain network with size *n*, the *n*×*m* matrix *B* = (*b*_1_, *b*_2_,…, *b_m_*) is an observation matrix of the *m* dimensional random vector 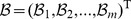. The covariance matrix of 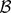 can be estimated by its observation matrix *B*. Denote the covariance matrix of *B* as 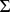, then



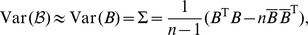
where 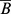 is the column mean vector of *B*, *n* is network size. It is noted that 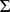 is just the unbiased estimator of 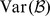 [48]. Based on the above notations, we have a stochastic form of *I* ^score^ as 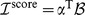. The variance of 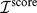 can be estimated by



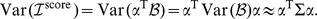



To determine the unique optimal vector 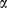, we restrict 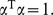 Thus, 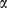 can be determined through the following constrained extremal problem:



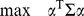


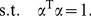
(2)


To solve the optimization problem (2), by the Lagrangian multiplier method, we construct the following Lagrangian function.
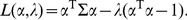
(3)


And let



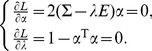
(4)where *E* is the identity matrix. It follows from Eq.(4) that 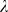 and 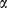 are just the eigenvalue and eigenvector of matrix 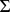. Under Eq.(4), 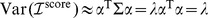. Therefore, the optimal 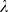 and 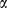 are just the biggest eigenvalue and the corresponding unit eigenvector of 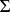. Denote the eigenvalues of 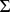 as 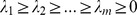, then the optimal 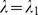. From the theory of the PCA, the ratio 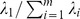 can reflect the contribution of 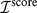, or how much information in 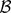 can be extracted by 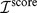.

So far we have determined 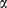. For a concrete network, replacing *b_j_* in Eq.(1) with concrete values, one determines the observation of 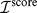 as *I* ^score^. Finally, the nodes in the network can be ranked according to *I* ^score^. Nodes with larger *I* ^score^ values are more structurally important. Based on *I* ^score^ and some well-defined distances, such as the well-known Euclidean distance, the *n* nodes can be classified into several clusters, where nodes in the same cluster are similarly important.

To sum up, for a network with *n* nodes, the procedures of the proposed measure are as follows.

1) Detect 2, 3 and 4-node network motifs in the network.

2) Count the occurrences of each node in *m* types of motifs, and derive a *n*×*m* matrix *A*.

3) Perform data processing on *A*, such as weighting and standardizing matrix *A*, then we obtain a matrix *B*. Compute the covariance matrix 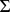 of *B*.

4) For 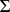, compute the biggest eigenvalue 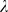 and the corresponding unit eigenvector 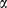.

5) Compute *I* ^score^ according to (1) and rank the *n* nodes accordingly.

### An illustrative example

**Figure 2:**
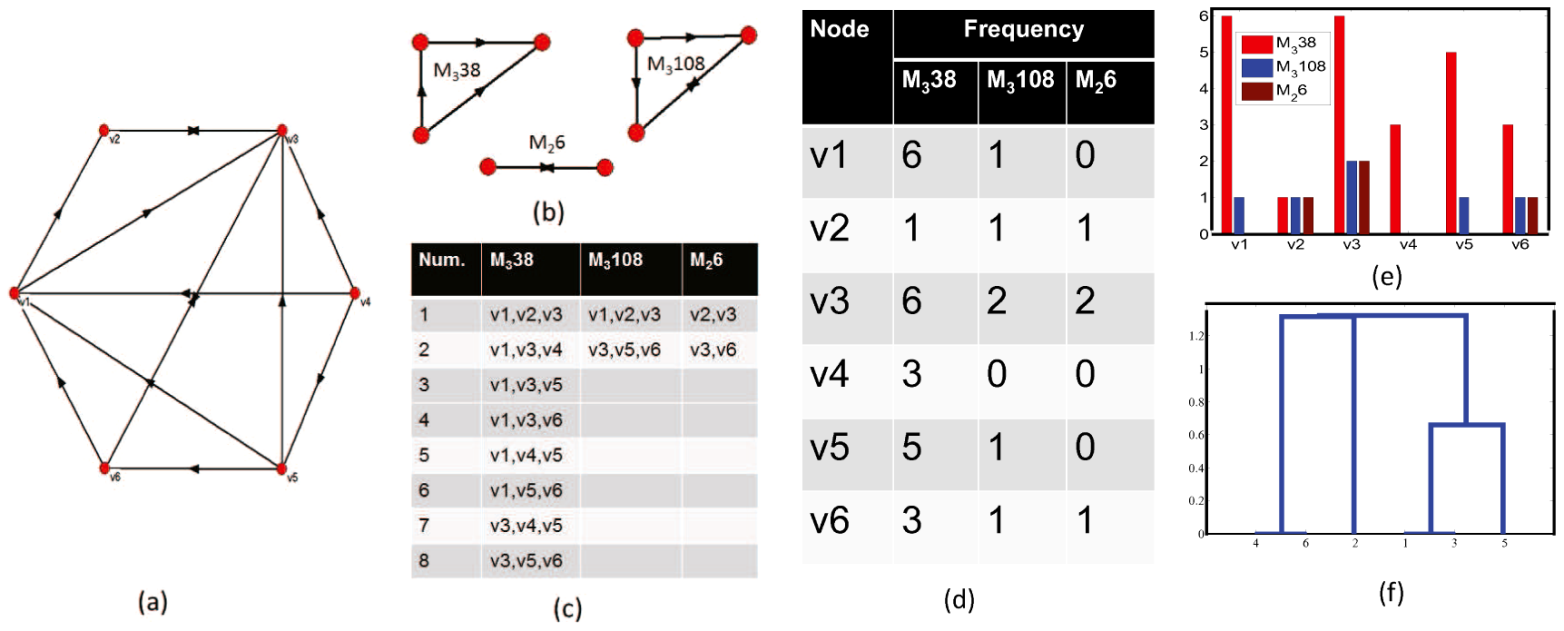
An illustrative example. (a) A simple network with six nodes. (b) Subgraphs that are assumed to be motifs in network (a). (c) Members that compose the three types of motifs. (d) Appearances of nodes in each motif as shown in panel (b). (e) Frequency histograms for the six nodes. (f) Cluster analysis reveals that the six nodes can be remarkably classified into three classes. *v*_1_, *v*_3_, *v*_5_ are the most important nodes, and *v*_2_ forms the least important group, *v*_4_, *v*_6_ form another group, which is more important than *v*_2_.

To illustrate the procedures of the proposed method, we give a simple example. The simple artificial network contains 6 nodes, and the topology of the network is shown in Fig. 2 (a). Suppose there are three motifs in the network, namely, *M*_3_38, *M*_3_108, *M*_2_6, as shown in Fig. 2(b). Fig. 2(c) lists the members of the three motifs. Occurrences of nodes in each motif are summarized in Fig. 2(d). As we see, the occurrences of *M*_3_38, *M*_3_108, *M*_2_6 are 8, 2 and 2, respectively. Therefore, the weights of *M*_3_38, *M*_3_108, *M*_2_6 are 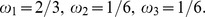 Subsequently, we derive matrix *B* and its covariance matrix 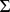.
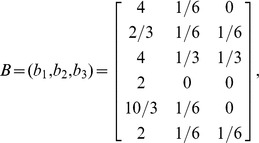


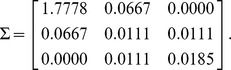



The eigenvalues of 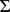 are 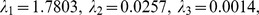 and the unit eigenvector corresponding to 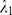 is:



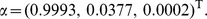



Thus, we have
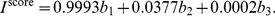
(5)


The contribution of *I* ^score^ is 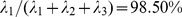. That is, 98.50% information that contained in *b*_1_, *b*_2_, *b*_3_ can be extracted by *I* ^score^. Therefore, *I* ^score^ can optimally rank the 6 nodes. Substitute *b*_1_, *b*_2_, *b*_3_ in matrix *B* into Eq.(5), we have
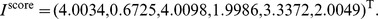



From *I* ^score^, the third value is the biggest. Therefore, we can judge that node *v*_3_ is the most important one, and then *v*_1_, the least important node is *v*_2_. If one simply considers the total occurrences of a node in all the motifs, then *v*_2_ and *v*_4_ would be treated as equally important. Whereas, from the proposed method, *v*_4_ is more important than *v*_2_, which is reasonable in that the occurrences of *M*_3_38 are significantly more frequent than the other motifs. Based on *I* ^score^ and through cluster analysis, the six nodes can be classified into three clusters, where *v*_1_, *v*_3_, *v*_5_ are members of the most important cluster; *v*_4_, *v*_6_ are members of the less important cluster; while *v*_2_ is the single member of the unimportant cluster.

### Data descriptions

The five real-world biological networks include the C. Elegans Neural (CEN) network [50], [51], the E. Coli Transcriptional (ECT) regulatory network from the RegulonDB database [52], the Yeast Transcriptional (YT) regulatory network [53], the Drosophila Developmental Transcriptional (DDT) network [13], and the Human Signal Transduction (HST) network [13]. We note that the investigated networks are with high quality and have been frequently used as models to detect network motifs [9], [11]–[13].

Simple statistical indexes for the five networks are summarized in Tab. 1. Numbers of nodes for these networks range from 119 to 1706. Numbers of edges range from 306 to 3870. The five networks are with abundant network motifs, such as the FFL *M*_3_38, *M*_3_46, the bi-fan *M*_4_204. It is noted that, we have considered all 2, 3-node motifs, but for simplicity, we have only considered three 4-node motifs: *M*_4_204, *M*_4_328 and *M*_4_904. There are totally 199 connected 4-node subgraphs, and there are many 4-node motifs in the five networks. For example, in the CEN and ECT, there are seven 4-node motifs. Since the bi-fan *M*_4_204 and the bi-parallel *M*_4_904 have been frequently investigated under various context [9], they are common motifs in many different real-world networks [11], and the 4-node chain *M*_4_328 may play crucial roles in signal transduction pathways, we will only consider these three 4-node motifs. From Tab. 1, the CEN has the most abundant of motifs. Subgraph *M*_2_6 is only a motif in the CEN and ECT, and the actually numbers are 233 and 10, respectively. The *M*_4_328 is only a motif in the HST, the actual number is 1570. There are no 3-node motifs in the HST. Whereas, for most of the networks, the FFL and bi-fan are motifs. The YT only consists of the FFL and bi-fan.

**Table 1:** Statistical indexes for the five directed biological networks.

Network	CEN	ECT	DDT	HST	YT
Node	280	1706	119	227	685
Edge	2194	3870	306	312	1052
Ave. in-degree	7.8357	2.2685	2.5714	1.3744	1.5358
Ave. out-degree	7.8357	2.2685	2.5714	1.3744	1.5358
Ave. total degree	15.6714	4.5369	5.1428	2.7489	3.0715
Ave. *I^score^*	5.6753	35.9339	2.0367	12.3849	7.2407
*M*_2_6	233	10	-	-	-
*M*_3_38	1453	1196	174	-	62
*M*_3_46	552	226	26	-	-
*M*_3_108	385	-	16	-	-
*M*_3_110	175	-	-	-	-
*M*_3_238	48	-	-	-	-
*M*_4_204	2274	29535	-	280	1812
*M*_4_328	-	-	-	1570	-
*M*_4_904	2253	-	-	275	-

“-” denotes no such item.

## Results

### Identifying important nodes in the five networks

Following the procedures as the illustrative example, one can obtain the order factor for each network. Noted that the occurrences of different motifs have different order of magnitude, we have performed standardized transformations to matrix *B*. Moreover, we denote the columns of matrix *B* as the vector *b_i_j*, where *i* and *j* have the same meaning as that in *M_i_j*.

The *I* ^score^ for the five networks are obtained as follows.
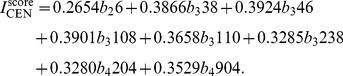
(6)

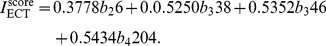
(7)

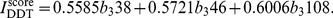
(8)

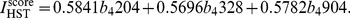
(9)

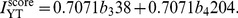
(10)


Replacing *b_i_j* with concrete values in matrix *B* for each network, one obtains the importance score for each node. Average *I* ^score^ values for the five networks are shown in Tab. 1. Based on *I* ^score^, we can characterize the node importance and classify the nodes for each network via cluster analysis. The basic idea of cluster analysis is as follows [48]. According to *I* ^score^, the Euclidean distance between any two nodes can be obtained. Firstly, two nodes with the shortest distance are merged as one group, each of the rest nodes forms a group. Then, one merges node groups via the single linkage method, until all nodes are finally merged into one cluster. This cluster processes can be mimicked by a dendrogram. From cluster analysis, one can classify nodes into groups, with similar important nodes in the same group. Furthermore, from the dendrogram, one can intuitively get some knowledge about the structural features of the network.

**Figure 3:**
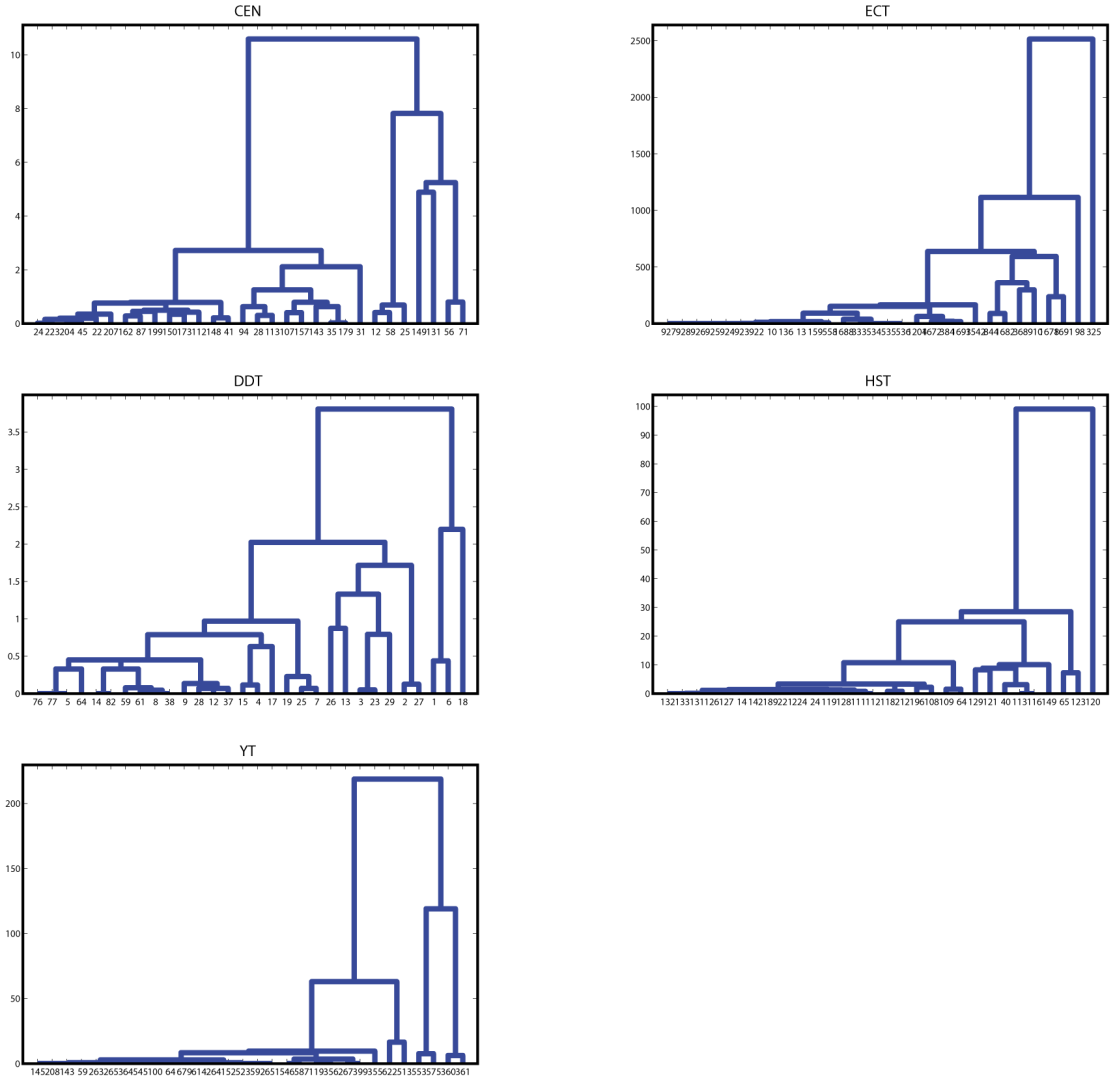
Cluster analysis for the identified top-30 nodes in the five networks based on the *I* ^score^.

Fig. 3 shows the dendrogram for the top-30 nodes of the five networks. We can see that these nodes can be roughly classified into three or four groups, detailed information of the top-30 nodes in the CEN, ECT, YT and their corresponding rankings by the other methods are summarized in Tabs. 2–4. The corresponding information for the DDT and HST are shown in Table S1 and S2. In each table, we have shown the in and out-degree as well as their rankings by the other methods. Here, *R*_total_ is based on the total degree, *R*_p_ is based on the PageRank, *R*_mc_ is based on the motif centrality, and *R*_bet_ is based on the betweenness. The motif centrality only considers the FFL, since there are no such motif in the HST, it fails to work in the HST. For each network, the last group contains the largest amount of nodes, while the most important group *G*_1_ contains only one to three nodes. From Fig. 3, for the five biological networks, only a few nodes are far more important than the others. There are clear hierarchical structures in these networks, which indicates that the proposed measure may also act as an effective hierarchical index.

**Table 2:** Clusters, members, rankings and statistical characteristics of the identified top-30 nodes in the CEN.

Group	Node	*I* ^score^	Out-deg.	*R*_out_	In-deg.	*R*_in_	*R*_total_	*R*_p_	*R*_mc_	*R*_bet_
*G*_1_	12:AVER	54.90	18	11	33	6	10	126	6	22
	58:AVBR	54.50	15	14	38	4	8	119	4	28
	25:AVEL	53.81	16	13	36	5	9	129	5	21
*G*_2_	149:AVDR	45.99	24	7	33	6	6	64	2	34
	131:AVDL	41.11	19	10	27	7	11	106	12	49
	56:AVBL	35.86	20	9	40	3	3	58	1	18
	71:AVAL	35.07	37	2	53	1	2	28	3	2
*G*_3_	94:AVJR	24.48	12	17	18	13	21	114	7	96
	28:AVAR	23.85	49	1	49	2	1	13	8	1
	113:AIBR	23.55	11	18	25	9	16	143	11	42
	107:DVA	22.30	35	3	19	12	7	24	9	7
	157:PVCL	21.90	32	4	27	7	4	32	10	5
	143:PVCR	21.11	32	4	26	8	5	16	13	3
	35:RICR	20.48	8	21	14	17	29	173	36	39
	179:ADAL	20.46	14	15	8	23	29	97	15	102
	31:RICL	18.35	12	17	11	20	28	130	36	56
	148:AVL	15.63	12	17	15	16	24	83	19	58
	41:ADEL	15.42	26	5	5	26	20	6	28	54
	204:PVNL	14.63	19	10	8	23	24	12	18	70
	45:RIAL	14.58	15	14	27	7	13	117	20	19
	24:AIBL	14.43	13	16	24	10	15	66	15	11
	223:AVG	14.42	17	12	5	26	29	4	14	69
	22:RIAR	14.22	18	11	26	8	12	108	17	10
	207:ASHR	13.88	13	16	8	23	30	60	30	119
	162:AVJL	13.12	14	15	21	11	17	25	16	31
	87:RMGR	12.84	14	15	8	23	29	23	40	53
	199:ADLR	12.38	15	14	2	29	34	77	23	146
	150:HSNR	11.89	25	6	16	15	14	8	22	8
	173:PVNR	11.55	22	8	11	20	19	15	23	36
	112:RIML	11.13	12	17	16	15	23	139	21	73

*R*_in_ and *R*_out_ represent the rankings by the in and out-degree. *R*_total_ and *R*_p_ represent the results from the total degree and PageRank [21]. *R*_mc_ and *R*_bet_ denote the results from the motif centrality [25] and betweenness. Similarly hereinafter.

**Table 3:** Clusters, members, rankings and statistical characteristics of the identified top-30 nodes in the ECT.

Group	Node	*I* ^score^	Out-deg.	*R*_out_	In-deg.	*R*_in_	*R*_total_	*R*_p_	*R*_mc_	*R*_bet_
*G*_1_	325:CRP	6643.21	496	1	1	13	1	2	2	6
*G*_2_	98:FNR	4128.98	295	2	3	11	2	4	1	2
*G*_3_	844:arcA	3014.10	173	6	1	13	6	6	18	9
	1682:IHF	2924.94	219	4	0	14	4	3	3	55
	368:fis	2564.89	226	3	2	12	3	1	7	8
	910:narL	2267.63	121	8	2	12	8	18	5	18
	1678:H-NS	1675.38	186	5	0	14	5	7	4	55
	1691:narP	1438.18	49	17	0	14	17	39	41	55
*G*_4_	1542:cra	801.16	78	12	1	13	12	17	23	21
	1204:lrp	637.09	104	9	3	11	9	14	8	3
	1672:FlhDC	574.76	80	11	0	14	11	12	41	55
	384:fur	555.05	128	7	3	11	7	5	6	4
	1693:NsrR	538.26	83	10	0	14	10	11	26	55
	1688:ModE	386.47	46	18	0	14	18	20	25	55
	333:cysG	348.34	0	52	8	6	43	134	38	55
	534:nirB	348.34	0	52	8	6	43	134	38	55
	535:nirC	348.34	0	52	8	6	43	134	38	55
	536:nirD	348.34	0	52	8	6	43	134	38	55
	159:pflB	257.65	0	52	6	8	45	134	38	55
	558:pdhR	251.49	41	20	3	11	19	27	17	15
	13:gadX	234.85	27	29	13	1	22	30	12	5
	10:gadA	218.98	0	52	11	3	40	134	28	55
	922:nrfA	218.77	0	52	7	7	44	134	38	55
	923:nrfB	218.77	0	52	7	7	44	134	38	55
	924:nrfC	218.77	0	52	7	7	44	134	38	55
	925:nrfD	218.77	0	52	7	7	44	134	38	55
	926:nrfE	218.77	0	52	7	7	44	134	38	55
	927:nrfF	218.77	0	52	7	7	44	134	38	55
	928:nrfG	218.77	0	52	7	7	44	134	38	55
	136:lpd	207.41	0	52	7	7	44	134	36	55

**Table 4:** Clusters, members, rankings and statistical characteristics of the identified top-30 ranked nodes in the YT.

Group	Node	*I* ^score^	Out-deg.	*R*_out_	In-deg.	*R*_in_	*R*_total_	*R*_p_	*R*_mc_	*R*_bet_
*G*_1_	553:STE12	489.54	71	1	0	11	1	1	10	19
	575:TEC1	482.02	44	3	0	11	3	7	10	19
*G*_2_	360:MSN2	363.05	35	6	0	11	6	14	10	19
	361:MSN4	356.90	32	7	0	11	7	16	10	19
*G*_3_	622:YAP1	138.11	38	4	0	11	4	6	10	19
	513:SKN7	121.70	21	13	0	11	12	19	10	19
*G*_4_	355:MIG1	58.87	26	9	0	11	9	4	7	19
	546:SSA4	49.23	0	32	4	7	28	100	10	19
	587:TKL2	49.23	0	32	4	7	28	100	10	19
	119:CTT1	45.81	0	32	6	5	26	100	10	19
	356:MIG2	45.79	12	20	0	11	20	31	9	19
	267:HSP78	42.39	0	32	4	7	28	100	10	19
	399:PGM2	42.39	0	32	6	5	26	100	10	19
	614:UME6	34.30	38	4	0	11	4	3	5	19
	264:HSP12	33.50	0	32	4	7	28	100	10	19
	152:DOG2	32.82	0	32	4	7	28	100	10	19
	523:SOD2	32.82	0	32	5	6	27	100	10	19
	592:TPS1	32.82	0	32	4	7	28	100	10	19
	651:YLR042C	32.82	0	32	4	7	28	100	10	19
	64:CAR2	30.08	0	32	6	5	26	100	10	19
	679:DAL81-DAL82	28.72	8	24	0	11	24	61	10	19
	263:HSP104	25.98	0	32	3	8	29	100	10	19
	265:HSP26	25.98	0	32	3	8	29	100	10	19
	364:MUC1	25.98	0	32	5	6	27	100	10	19
	545:SSA3	25.98	0	32	3	8	29	100	10	19
	100:CLN1	25.96	0	32	5	6	27	100	9	19
	59:BNI5	25.30	0	32	2	9	30	100	10	19
	143:DDR48	25.30	0	32	3	8	29	100	10	19
	145:DHH1	25.30	0	32	2	9	30	100	10	19
	208:GAT4	25.30	0	32	2	9	30	100	10	19

### Functional characteristics of the top-ranked nodes

In the following, for the CEN, ECT and YT, we discuss whether the identified structurally top-ranked nodes are functionally important.

For the CEN, the identified top-30 nodes are shown in Tab. 2. The top-7 nodes are AVER, AVBR, AVEL, AVDR, AVDL, AVBL and AVAL, which are all command interneurons. Additionally, the AVAR, PVCL and PVCR are another three command interneurons, which are all top-ranked. The AVAs, AVBs, AVDs, and PVCs are four bilaterally symmetric interneuron pairs with large diameter axons that run the entire length of the ventral nerve cord, and providing inputs to the ventral cord motor neurons. The AVAs locate at the lateral ganglia of head of the C. elegans, functioning as driver cell for backward locomotion [54]. The AVEs can drive backward movement of the animal along with AVAs, AVDs and A-type motor neurons [54]. The AVDs function as touch modulator for backward locomotion induced by head-touch. The PVCs are ventral cord interneurons, a harsh touch defect can be caused in the absence of PVC neurons [54]. From Tab. 2, the AVER has the largest *I* ^score^ value 54.90, the in and out-degree of AVER are 33 and 18, which are not the largest. However, from our investigation, the AVER is the most important nodes in the CEN, which demonstrates that the *I* ^score^ is different from the degree measures. The PageRank fails to identify most of the command interneurons as even among the top-50 level. The betweenness ranks many of the command interneurons out of the top-20 level. The results for the CEN indicate *I* ^score^ can help to identify the actual important nodes.

For the ECT, the identified top-30 nodes are shown in Tab. 3. In 2003, Martínez-Antonio et al. [55] identified global regulators in an ECT network. There are 18 global regulators in the network, namely, CRP, IHF, FNR, fis, arcA, lrp, hns, narL, ompR, fur, phoB, cpxR, soxR, soxS, mlc, cspA, rob, purR. Among which, the CRP, FNR, IHF, fis, arcA, narL, lrp are seven key regulators, which can regulate the expression of 51% of genes in E. coli [55]. From *I* ^score^, eight of the top-12 nodes (CRP, FNR, arcA, IHF, fis, narL, lrp, fur) are global regulators. The in-degree ranks most of the eight global regulators at the tail. The out-degree and total degree rank most of the eight global regulators at the top-10 level. According to the PageRank, motif centrality and betweenness, 2, 1 and 3 of the identified top-ranked global regulators are out of the top-10 level. The global regulator CRP is the most important nodes, which represents the cAMP receptor protein. The CRP can regulate cAMP, and genes regulated by the CRP are mostly involved in energy metabolism [56]. The CRP has the largest out-degree 496. But its in-degree is only 1. Though 280: csgE has the second largest in-degree 12, it is not top-30 ranked. From Tab. 3, the top-30 nodes can be classified into four clusters. The unimportant cluster contains the largest amount of nodes. The first three clusters are almost all global regulators. The observations from the ECT indicate that the proposed measure can help to find global regulators.

For the YT, the top-30 nodes are shown in Tab. 4. STE12 and TEC1 are two most important nodes, with the *I* ^score^ values 489.54 and 482.02, with the out-degree 71 and 44, and with the in-degree both 0. STE12 and TEC1 are two transcription factors. It has been reported that the STE12 controls two distinct developmental programs of mating and filamentation, therefore, it is a key regulator of cell fate determination [57]. Although the TEC1 gene has been reckoned as involving in the activation of expression of Tyl and the adjacent genes, it is not essential in the control of mating or sporulation processes [58]. It is intriguing to clarify why TEC1 is so frequently involved in network motifs and acts as building blocks of the YT network. From the results of the out-degree, total degree, PageRank, motif centrality and betweenness, most of the nodes in *G*_4_ are equally important, and thus have great differences from *I* ^score^.

### Performance evaluation based on ROC curves

To evaluate the performance of *I* ^score^, we perform ROC analysis. ROC curve is frequently used to evaluate the performance of a new test in the field of signal processing and medical diagnostic tests [59]. For a network with *n* nodes, the procedures of ROC analysis are as follows. Suppose the nodes can be classified into two groups: important and unimportant, and we know the actual classification. For a new index, the *n* nodes are with values in the interval [*a*, *b*], for any threshold value 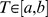, one can reclassify the *n* nodes into two classes. Comparing the actual classification with the new classification, several indexes can measure the accuracy of the new index, which are defined as follows [59].
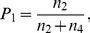
(11)

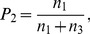
(12)

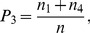
(13)where *n*_2_ denotes the number of false positive nodes, which are considered important in the new classification but actually unimportant. *n*_4_ gives the number of true negative nodes, where the nodes are both unimportant in the two classifications. Similarly, *n*_1_ and *n*_3_ denote the number of true positive and false negative nodes, respectively. *P*_1_, *P*_2_ are therefore called false and true positive rates, respectively. *P*_3_ is called the accuracy of the new index. Given a *T*, one obtains a point (*P*_1_, *P*_2_). For 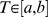, plotting the corresponding points in two dimensional coordinate system, we derive the ROC curve. The area under the curve (AUC) of ROC equals the probability that a classifier will rank a randomly chosen positive instance higher than a randomly chosen negative one [59], which can reflect the identification accuracy of the new index. The larger AUC, the more accurate of the index. Furthermore, the point in the upper left corner of a curve corresponds to the optimal threshold *T*, which gives the new classification of nodes with the highest *P*_3_.

**Figure 4:**
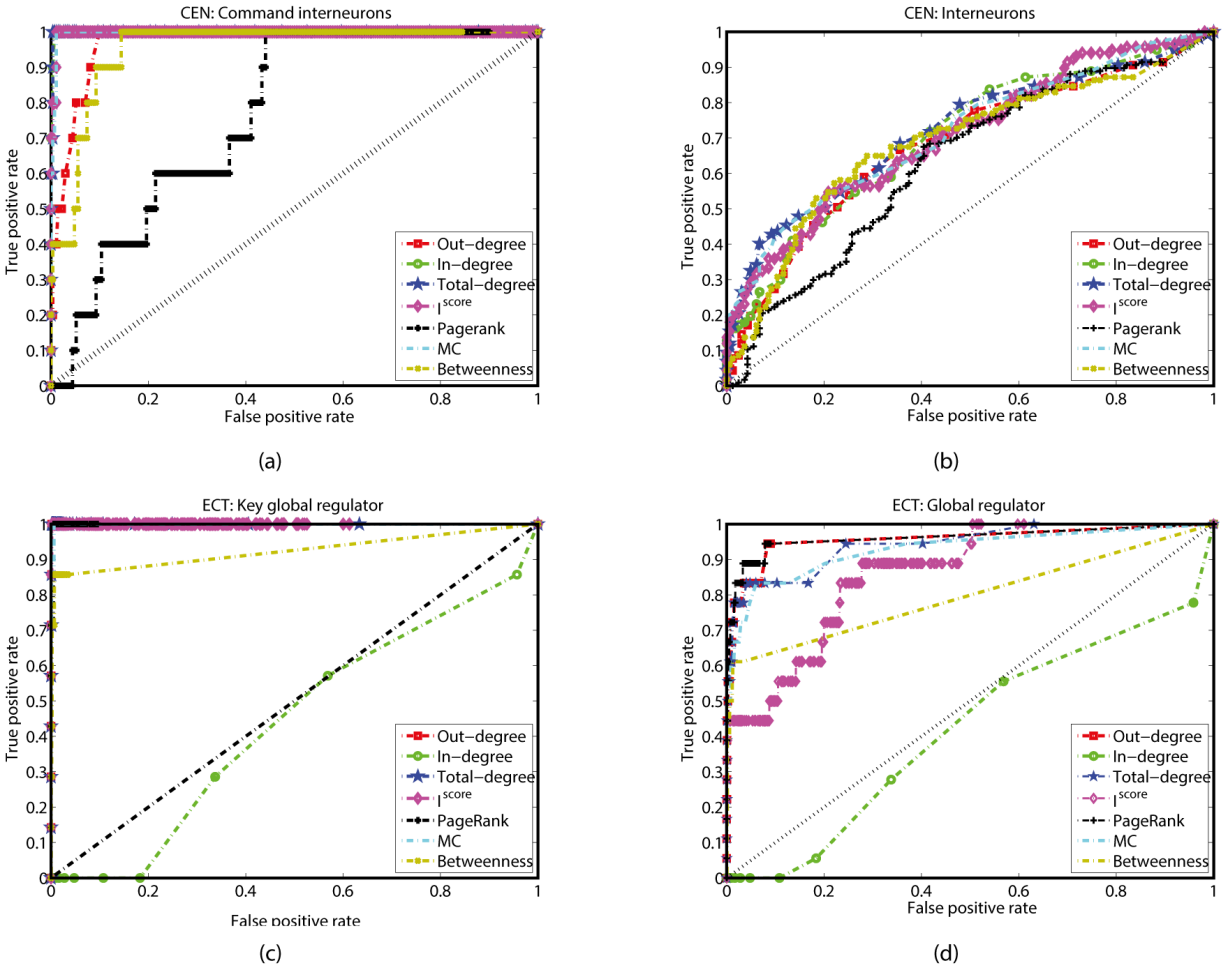
ROC curves based on the available information in the CEN and ECT. (a) Performance of different indexes in identifying (a) the 10 command interneurons in the CEN, (b) the 117 interneurons in the CEN, (c) the 7 key global regulators in the ECT, (d) the 18 global regulators in the ECT.

Hereinafter, based on the available information of some of the investigated networks and ROC curves, we evaluate the performance of *I* ^score^ and the other indexes. In the following, for simplicity, we transform node ranks into fractional ones (range in (0, 1]). For nodes with rank *k*, its fractional ranks are the ratio of the number of nodes with ranks no more than *k* to *n*. Obviously, nodes with smaller fractional ranks are more important. For the CEN, on one hand, we have mentioned that the 10 command interneurons are known to be very important. If we take them as important nodes, one derives the ROC curves for each index, as shown in Fig. 4(a). From Fig. 4(a), the in-degree, total-degree, *I* ^score^ and motif centrality all can well identify the command interneurons, the AUC (trapezoidal method) for these indexes are 0.9991, 0.9985, 0.9974, 0.9967, which are all above 0.99. The *I* ^score^ is a little better than the motif centrality. The out-degree, PageRank and betweenness are all worse than the other indexes. On the other hand, neurons in the C. Elegans can be classified into interneurons, motor neurons, sensory neurons, where 117 neurons function as interneurons. If we take the 117 interneurons as important nodes, one obtains another ROC curve for each index, as shown in Fig. 4(b), where all the seven measures have roughly similar performance. The *I* ^score^ is a little better than the out-degree, in-degree, PageRank and betweenness. For the ECT, there are 7 key and totally 18 global regulators, which are actually important in the network. If we take the 7 key global regulators and 18 global regulators as actually important nodes, we derive two ROC curves for each index, as shown in Fgs.4(c) and (d). In Fig. 4(c), the AUC for the seven indexes are 0.9996, 0.4385, 0.9996, 0.9997, 0.9983, 0.9987 and 0.9239. Except the in-degree and betweenness, all the indexes can well identify the key global regulators. *I* ^score^ is a little better than the other indexes. From Fig. 4(d), the out-degree, total-degree, PageRank and motif centrality are with quite large AUC. The AUC for the *I* ^score^ is 0.8628, which is only higher than that for the in-degree and betweenness, however, when *T* = 0.0036, the *I* ^score^ can classify the nodes in the ECT with *P*_3_ = 99.30%.

**Figure 5:**
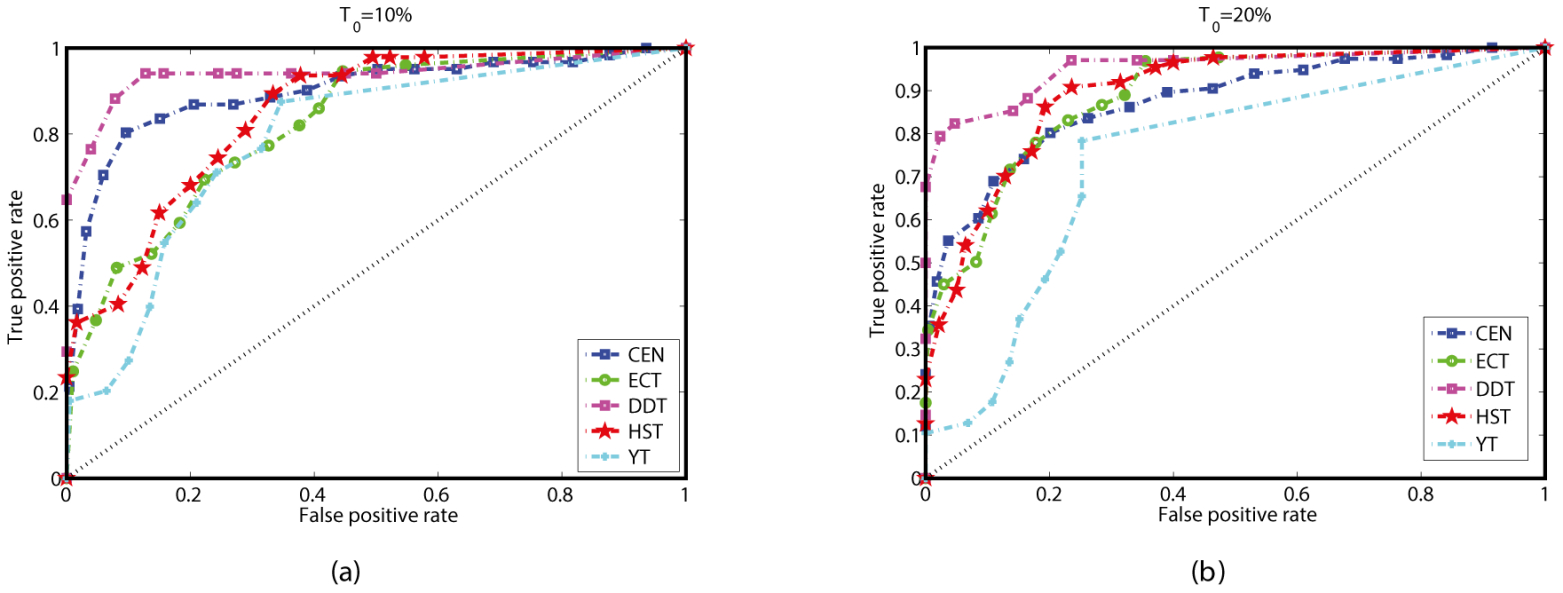
Evaluation of *I* ^score^ via ROC curves with composite reference standards for the five networks. (a) *T*_0_ = 10%. A node is defined as important if either its rankings by the in, out, total degree, PageRank, motif centrality or the betweenness are at the top-*T*_0_ level. (b) Similarly to (a), but with *T*_0_ = 20%.

For many biological networks, the actual classifications, known as gold standards, are not available. Fortunately, researchers have proposed several methods to evaluate the new test, such as constructing composite reference standards from available multiple tests [60], [61]. A single ranking from either the in, out, total degrees, PageRank, motif centrality or betweenness is imperfect and can not act as a gold standard. Subsequently, for each network, we construct a composite reference standard based on the six rankings (Five in the HST), and evaluate the accuracy of *I* ^score^. Specifically, in the composite reference standard, a node is defined as important if either one of the six rankings is among the top-*T*_0_, where *T*_0_ is a threshold, which can be taken as 10%, 20% and so on. Thus, given a *T*_0_, we derive a dichotomous reference classification of nodes in the network, either positive (important) or negative (unimportant). According to the ranking from the *I* ^score^, we take several threshold values *T* to reclassify nodes, and finally derive the ROC curves for each network, as shown in Fig. 5. Figs. 5(a) and (b) show the cases with *T*_0_ = 10% and *T*_0_ = 20%, respectively. In Fig. 5(a), the AUC for the five networks are 0.8977, 0.8237, 0.9406, 0.8499 and 0.7878, respectively. The points in the upper left corner of the ROC curves in Fig. 5(a) correspond to *T* = 20%, 5%, 10%, 10%, 5%, which lead to the highest *P*_3_. For example, for the DDT, when the top-10% nodes are classified as important ones, the classification from the *I* ^score^ has the best consistency with the reference classification, the *P*_3_ can achieve 94.96%. For *T*_0_ = 20%, the AUC for the five networks are 0.8740, 0.8884, 0.9521, 0.8955 and 0.7418, respectively. Under two different *T*_0_ and for different networks, the AUC are all above 0.74. Especially, in the DDT, the AUC is above 0.94, which indicates high identification accuracy of the proposed measure.

It is noted that, for the DDT, HST and YT, since we still do not know how many nodes are actually important, it is difficult to compare among different measures via ROC curves. We also note that the ROC analysis without gold standards may subject to bias of the composite reference standard. However, since the composite reference standards for the five networks are based on six or five existing measures, it is trustworthy to treat them as reference standards. In conclusion, ROC analysis indicates the proposed measure is a remarkable alternative index to identify structurally important nodes in directed networks.

### Topological neighborhoods of several special nodes

From the ROC analysis in the CEN and ECT, some measures are better than the *I* ^score^ in identifying the command interneurons or global regulators. Hereinafter, through the specific analysis on topological neighborhoods of several nodes, we further illustrate the merits of the proposed measure. According to *I* ^score^, some hubs may be not important, whereas some non-hub nodes may be identified as very important ones. There are many highly connected but not highly ranked nodes, such as 946: soxs in the ECT; 22: b-catenin and 68: fak in the HST; 209: GCN4 and 332: MBP1-SWI6 in the YT. Examples of nodes with low degrees but ranked at top-20 include 333: cysG and 534-536:nirB-nirD in the ECT; 546: SSA4 and 587:TKL2 in the YT.

**Figure 6:**
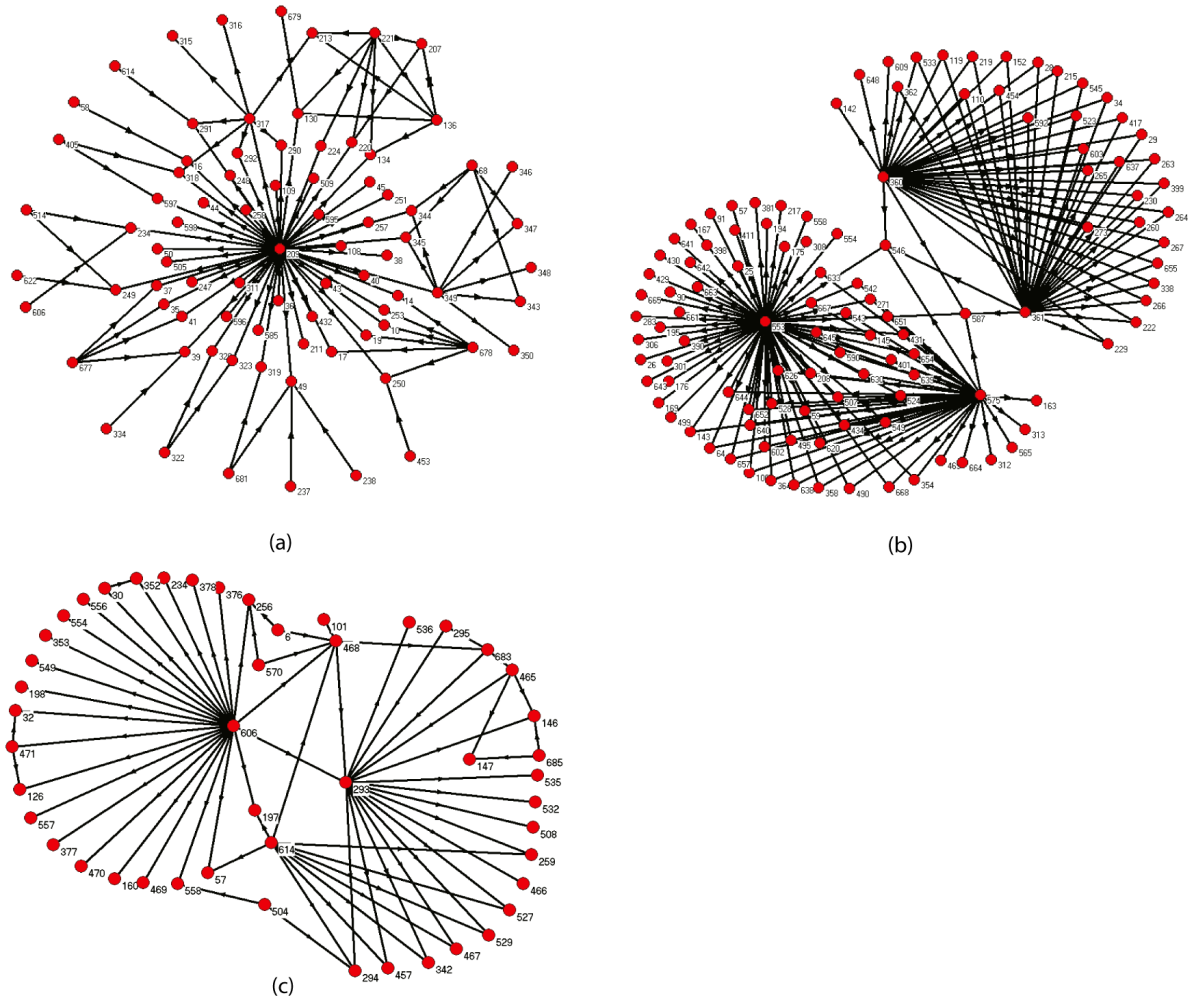
Topological neighborhoods of several nodes. (a) Topological neighborhood of a hub but not top-ranked node: node 209 in the YT. (b) Topological neighborhood of a non-hub but top-ranked node: node 546 in the YT. (c) Topological neighborhood of a not top-ranked node but with the highest betweenness: node 293 in the YT.

In the following, we take node 209 and 546 in the YT as two representative examples. Node 209 has out-degree 53 and in-degree 0, which is the second most important node according to the out and total degree, while its ranking is 62 according to *I* ^score^. Node 546 is with the in and total degree 4, the ranking is 28 according to the total degree, but it is ranked as the eighth most important node by the *I* ^score^. Figs. 6 (a) and (b) visualize the topological neighborhoods of the two nodes with their nearest and second nearest neighbors. From the topological neighborhoods of the two nodes, there are 81 nodes involved in the neighborhood of node 209, which are connected by 111 directed edges that centered at node 209, while 114 nodes and totally 182 directed edges consist of the neighborhood of node 546. The connection density of the neighborhood of node 209 is much lower than node 546. Moreover, from Fig. 6 (b), one can easily see that node 546 is directly regulated by four hub nodes and acts a bridge or bottleneck of the topological neighborhood. More importantly, the four hub neighbors of node 546 are just the top-4 nodes. Though node 209 can regulate 53 nodes, but its neighbors are neither hubs nor important nodes. Furthermore, node 546 involves in 1203 bi-fan subgraphs in its topological neighborhood, while there are only 39 such subgraphs for node 209, which indicate node 546 may play more functional roles in the system. Therefore, node 546 may be more important than 209. Finally, from the roles of biological functions, node 209 represents GCN4. It has been found that the GCN4 gene is conserved in S. cerevisiae, K. lactis, and E. gossypii [62]. SSA4 is widely conserved in human, chimpanzee, Rhesus monkey, dog, cow, mouse, rat, chicken, zebrafish, fruit fly, C. elegans, S. cerevisiae, and A. thaliana [62]. The cross species conservation of a gene indicates that it has been maintained by evolution despite speciation. It has been widely believed that mutation in a highly conserved gene can lead to a non-viable life form, or a form that is eliminated through natural selection [62], [63]. SSA4 is more widely cross species conserved, which also indicates that SSA4 is more important than GCN4. Summing up, it is sufficient that the non-hub node 546 is actually more important than the hub node 209.

From the above analysis, it seems that node 546 similarly functions as nodes with high betweenness in undirected networks. However, we note that there are great differences between directed and undirected networks. In the YT, the node 209 has only 53 outgoing edges and the node 546 has only 4 ingoing edges, the betweenness [19] of the two nodes are both zeros, which are the least important nodes according to it. Therefore, the *I* ^score^ is different from the classical betweenness. Furthermore, since the YT is a directed network, the betweenness of 96.06%(658/685) nodes are zeros, it fails to act as an effective ranking measure. It is noted that node 293: IME1 has the largest betweenness in the YT, whereas, it is not highly ranked according to *I* ^score^. Fig. 6 (c) shows the topological neighborhood of node 293. Node 293 is with 5 ingoing and 13 outgoing edges, but it is not frequently involved in network motifs. In conclusion, from the topological neighborhoods of several concrete nodes, we can further conclude that the proposed measure has its merits.

## Discussion

Biological networks are typical real-world complex networks. It has been reported that a single measure is insufficient to distinguish lethal nodes clearly from viable ones in some biological networks [26], [64]. Therefore, it is intriguing to find some more effective measures to characterize node differences in biological networks. In this paper, based on the integration of the occurrences of each node in 2, 3 and some 4-node network motifs, we have proposed a new measure to characterize node importance in biological networks. Based on ROC curves and the analysis of the topological neighborhoods of several specific nodes, we have compared the obtained results with that from the degree, PageRank, motif centrality and betweenness.

In the CEN and YT, when the command interneurons, interneurons, key global regulators and global regulators are treated as actually important nodes, we compared the performance among different measures. The proposed measure has good performance in the two networks. The in-degree is good at identifying command interneurons in the CEN, but it is bad at finding global regulators in the ECT. The out-degree displays the contrary tendency as the in-degree. Though the PageRank can effectively identify the global regulators in the ECT, it is the worst measure in identifying command interneurons or interneurons in the CEN. Similarly, the betweenness is also not a good measure in the two networks. Therefore, the in-degree, out-degree, PageRank and betweenness are not robust indicators of important nodes in different networks. The *I* ^score^ provides an alternative robust measure for different types of biological networks.

**Figure 7:**
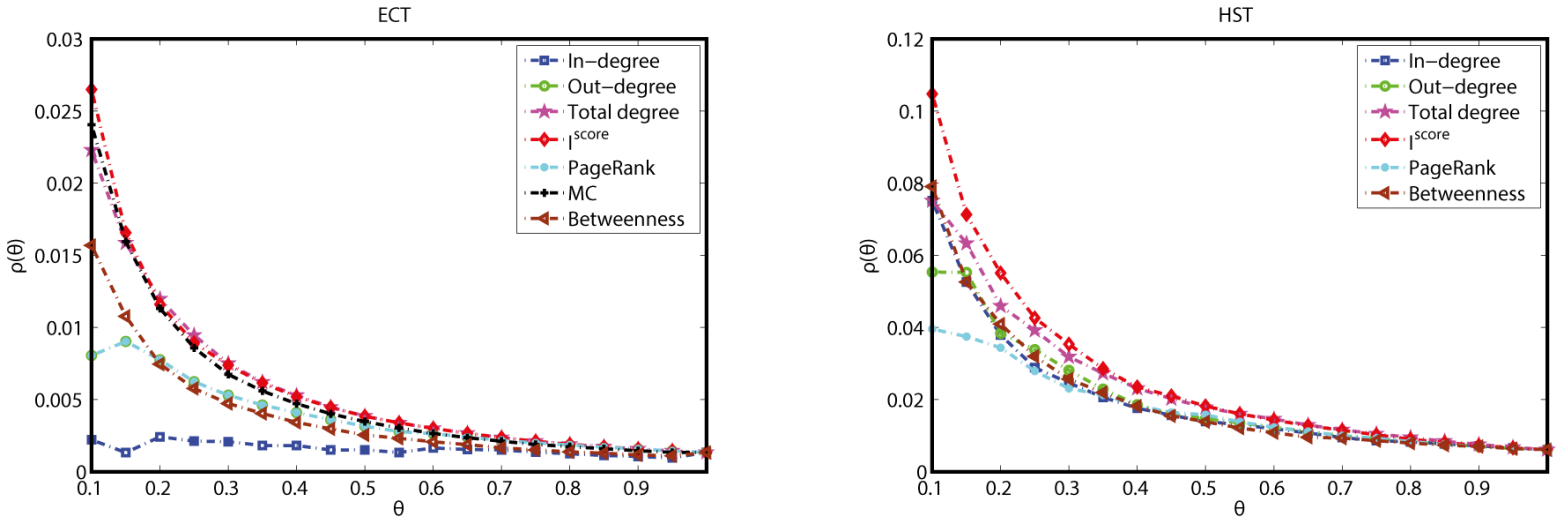
The curves of connectivity density 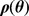 against 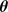 for different ranking measures in the ECT and HST.

Since the current knowledge on the five networks are limited, we note that it is still an open problem to further mining the advantages of the new measure. The number of command interneurons in the CEN and global regulators in the ECT are much fewer than the network sizes, the ROC analysis may suffer the effect of noise both in the interaction data and computation processes. We note that some other approaches may be used to further investigate the merits of the new measure, such as rich-cub analysis [8], [65]–[69]. For simplicity, we simply examine the connectivity densities among the same amount of top-ranked nodes according to different measures in the ECT and HST, as shown in Fig. 7. Here, 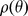 is defined as the ratio of the total actual number of edges to the maximum possible number of edges among the top-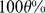 nodes. In Fig. 7, different curves correspond to different indexes. From Fig. 7, we can see that for many indexes, top-ranked nodes tend to be with higher connectivity densities than nodes ranked at the tail. The motif centrality fails to work in the HST, since the FFL is not a motif in such network. Moreover, comparing among different indexes, the *I* ^score^ is very good at finding the cluster with high connectivity densities. That is, the connectivity density among a few motif-rich nodes are higher than the same number of top-ranked nodes by the other indexes. For example, in the HST, the connectivity density among the top-10% (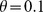) motif-rich nodes is above 0.10, while the top-10% large-degree nodes are with 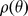 below 0.08.

It has been reported that many bio-molecular networks are disassortative, which have negative Pearson correlation coefficients (PCCs) [6]. For example, the PCCs of the CEN and YT are −0.0537 and −0.3496. The disassortativity indicates that large-degree nodes would connect with low-degree ones rather than with each others. Whereas, nodes with high *I* ^score^ involve in many network motifs. Motif-rich nodes tend to form small connected subgraphs. Thus, the *I* ^score^ may be helpful to find clusters with high connectivity density in disassortative networks.

Finally, we note that this paper only considers five real-world biological networks, it is intriguing to further investigate the performance of the *I* ^score^ in some artificial networks, such as artificial scale-free, small-world networks and networks with community structures. It is noted that for networks with large cliques at the periphery, nodes in the cliques may have very high *I* ^score^ values, and therefore, these nodes may be highly ranked. Therefore, for such networks, the identified highly ranked clusters are probably just the large cliques. We will further investigate the related questions in our future works.

## Conclusions

In this paper, based on network motifs and multivariate statistical analysis, we have proposed a novel measure to characterize node importance in directed biological networks. The new measure enable us to further mining undiscovered characteristics of nodes in directed biological networks. Through the new measure, we have investigated five real-world biological networks, which include a neural network, three transcriptional regulatory networks and one signal transduction network. These networks vary in sizes and link densities, and consist of various types of network motifs.

Based on the proposed measure, we have identified important nodes in the five networks. Our investigations suggest that the most important nodes in biological networks only take up a small fractions, but many of them are with important biological functions in real-world biological systems. Moreover, ROC analysis reveals that the proposed measure is a rather stable indicator of important nodes, and with very high prediction accuracy. Furthermore, the proposed measure can well characterize non-hub but very evolutionary conserved functional important nodes, and simultaneously, exclude hubs but not so functionally important nodes from the top rankings. Finally, we have discussed that the proposed measure may be used to reveal clusters with high connectivity density in disassortative networks. From these statistical analysis, we conclude that the proposed measure has some unique merits and it can be acted as an alternative network metric.

Although we have mainly investigated some directed biological networks, the proposed measure can be extended to some other networks, such as electrical networks, social networks. It is also noted that the proposed measure can be extended to involve more types of network motifs, but with the increasing of motifs, the computational complexity will be increased. Moreover, if the FFL is the unique network motif in a directed network, the proposed method will degenerate into the motif centrality [25]. Lastly, we note that this paper provides an alternative way to characterize node features, it is still an open problem to find more effective ranking measures for nodes in directed biological networks, since it is generally difficult to obtain the actual rankings and a single measure is often insufficient to perfectly characterize all nodes. The related researches can help us to identify the actual key nodes in real-world systems. Real-world implications of identifying the key nodes include the finding of network control and regulation targets. For example, we can explore disease-associated or essential genes in cellular networks [70]–[72] for pharmacological or re-engineering purpose.

## Supporting Information

Table S1 **Clusters, members, rankings and statistical characteristics of the identified top-30 ranked nodes in the DDT.**
(PDF)

Table S2 **Clusters, members, rankings and statistical characteristics of the identified top-30 ranked nodes in the HST.**
(PDF)
