# Supplementary material for: Identification of Important Nodes in Directed Biological Networks: A Network Motif Approach
Source: PLoS One. 2014 Aug 29;9(8):e106132. doi: 10.1371/journal.pone.0106132 (PMC4149525; doi:10.1371/journal.pone.0106132)
Supplement: Table S1 — Clusters, members, rankings and statistical characteristics of the identified top-30 ranked nodes in the DDT. (PDF) [file pone.0106132.s001.pdf]

Table S1: Clusters,members, rankings and statistical characteristics of the identified top-30 ranked nodes in the DDT.

Group	Node	I^{score}	Out-deg.	R_{out}	In-deg.	R_{in}	R_{total}	R_{p}	R_{mc}	R_{bet}
G_1	1	20.01	15	3	4	9	5	7	1	23
	6	19.57	17	1	3	10	4	2	1	28
	18	17.37	9	6	15	1	2	14	2	2
G_2	26	13.57	8	7	7	6	8	16	3	15
	13	12.69	10	5	8	5	6	9	4	5
	3	11.36	9	6	8	5	7	10	6	10
	23	11.32	3	12	11	3	9	40	5	24
	29	10.53	15	3	0	13	8	3	7	49
	2	8.81	15	3	7	6	3	5	9	9
	27	8.69	8	7	5	8	10	17	8	22
G_3	19	6.66	16	2	12	2	1	6	10	1
	25	6.44	7	8	6	7	10	19	10	13
	7	6.37	5	10	3	10	14	26	10	43
	15	5.40	6	9	3	10	13	27	11	34
	4	5.28	4	11	6	7	12	18	12	14
	17	4.66	9	6	10	4	5	12	13	7
	9	3.87	6	9	8	5	9	11	14	4
	28	3.74	0	15	6	7	16	62	14	49
	12	3.67	7	8	5	8	11	22	14	18
	37	3.60	13	4	1	12	9	1	14	30
	14	3.15	3	12	5	8	14	46	15	25
	82	3.15	0	15	6	7	16	62	15	49
	59	2.82	9	6	6	7	8	13	17	3
	61	2.74	4	11	3	10	15	32	16	26
	8	2.70	5	10	3	10	14	24	16	11
	38	2.70	3	12	3	10	16	35	16	38
	5	2.25	8	7	0	13	14	8	17	49
	76	2.25	0	15	6	7	16	62	17	49
	77	2.25	0	15	6	7	16	62	17	49
	64	1.92	8	7	4	9	11	25	19	19
