# Supplementary material for: Identification of Important Nodes in Directed Biological Networks: A Network Motif Approach
Source: PLoS One. 2014 Aug 29;9(8):e106132. doi: 10.1371/journal.pone.0106132 (PMC4149525; doi:10.1371/journal.pone.0106132)
Supplement: Table S2 — Clusters, members, rankings and statistical characteristics of the identified top-30 ranked nodes in the HST. (PDF) [file pone.0106132.s002.pdf]

Table S2: Clusters, members, rankings and statistical characteristics of the identified top-30 ranked nodes in the HST.

Group	Node	I^{score}	Out-deg.	R_{out}	In-deg.	R_{in}	R_{total}	R_{p}	R_{mc}	R_{bet}
G_1	120: MKK4	249.37	5	6	12	1	2	25	-	6
G_2	65:ERK	150.31	12	1	10	2	1	5	-	5
	123:MKK7	179.63	3	8	12	1	4	50	-	8
G_3	129:ASK1	114.55	6	5	5	6	6	21	-	3
	121:MKK5	106.32	9	2	7	4	3	10	-	11
	40:JNK	97.49	6	5	6	5	5	38	-	9
	113:JNK3	94.40	6	5	5	6	6	38	-	12
	116:JNK2	94.40	6	5	5	6	6	38	-	12
	149:p38beta	84.36	8	3	4	7	5	19	-	19
G_4	109:MSK2	59.39	3	8	4	7	9	69	-	28
	64:ERK1	57.87	6	5	6	5	5	26	-	17
	118:MKK2	47.15	2	9	4	7	10	62	-	15
	212:TAK1	46.36	4	7	2	9	10	32	-	21
	196:SHC	43.10	2	9	6	5	8	22	-	4
	108:MSK1	40.92	3	8	3	8	10	69	-	40
	24:B-Raf	37.60	3	8	2	9	11	44	-	7
	119:MKK3	36.33	3	8	2	9	11	39	-	22
	128:MEKK4	35.13	2	9	3	8	11	83	-	39
	111:MNK1	34.38	1	10	4	7	11	89	-	49
	112:MNK2	34.38	1	10	4	7	11	89	-	49
	14:ATF2	33.00	0	11	3	8	13	92	-	71
	142:NFAT4	33.00	0	11	3	8	13	92	-	71
	189:RNPK	33.00	0	11	3	8	13	92	-	71
	221: p53	33.00	0	11	3	8	13	92	-	71
	224: c-JUN	33.00	0	11	3	8	13	92	-	71
	126:MEKK2	31.87	3	8	1	10	12	61	-	38
	127:MEKK3	31.87	3	8	1	10	12	61	-	38
	131:MLK1	31.77	2	9	2	9	12	83	-	61
	132:MLK2	31.77	2	9	2	9	12	83	-	61
	133:MLK3	31.77	2	9	2	9	12	83	-	61
